# Synthesis and Evaluation of a Thermoresponsive Degradable Chitosan-Grafted PNIPAAm Hydrogel as a “Smart” Gene Delivery System

**DOI:** 10.3390/ma13112530

**Published:** 2020-06-02

**Authors:** Monika Ziminska, Jordan J. Wilson, Emma McErlean, Nicholas Dunne, Helen O. McCarthy

**Affiliations:** 1 School of Pharmacy, Queen’s University of Belfast, 97 Lisburn Road, Belfast BT9 7BL, UK; m.ziminska@qub.ac.uk (M.Z.); jwilson88@qub.ac.uk (J.J.W.); E.McErlean@qub.ac.uk (E.M.); 2 School of Chemistry and Chemical Engineering, Queen’s University of Belfast, Belfast BT9 5AG, UK; 3 School of Mechanical and Manufacturing Engineering, Dublin City University, Dublin 9, Ireland; 4 Centre for Medical Engineering Research, School of Mechanical and Manufacturing Engineering, Dublin City University, Dublin 9, Ireland; 5 Department of Mechanical and Manufacturing Engineering, School of Engineering, Trinity College Dublin, Dublin 2, Ireland; 6 Advanced Manufacturing Research Centre (I-Form), School of Mechanical and Manufacturing Engineering, Dublin City University, Glasnevin, Dublin 9, Ireland; 7 Advanced Materials and Bioengineering Research Centre (AMBER), Trinity College Dublin, Dublin 9, Ireland; 8 Advanced Processing Technology Research Centre, Dublin City University, Dublin 9, Ireland; 9 Trinity Centre for Biomedical Engineering, Trinity Biomedical Sciences Institute, Trinity College Dublin, Dublin 2, Ireland; 10 School of Chemical Sciences, Dublin City University, Dublin 9, Ireland

**Keywords:** hydrogel, thermoresponsive, hydroxyapatite, nanoparticles, drug delivery

## Abstract

Thermoresponsive hydrogels demonstrate tremendous potential as sustained drug delivery systems. However, progress has been limited as formulation of a stable biodegradable thermosensitive hydrogel remains a significant challenge. In this study, free radical polymerization was exploited to formulate a biodegradable thermosensitive hydrogel characterized by sustained drug release. Highly deacetylated chitosan and N-isopropylacrylamide with distinctive physical properties were employed to achieve a stable, hydrogel network at body temperature. The percentage of chitosan was altered within the copolymer formulations and the subsequent physical properties were characterized using ^1^H-NMR, FTIR, and TGA. Viscoelastic, swelling, and degradation properties were also interrogated. The thermoresponsive hydrogels were loaded with RALA/pEGFP-N1 nanoparticles and release was examined. There was sustained release of nanoparticles over three weeks and, more importantly, the nucleic acid cargo remained functional and this was confirmed by successful transfection of the NCTC-929 fibroblast cell line. This tailored thermoresponsive hydrogel offers an option for sustained delivery of macromolecules over a prolonged considerable period.

## 1. Introduction

Hydrogels are three-dimensional, crosslinked polymeric networks capable of absorbing large amounts of water or biological fluids [1]. This group of synthetic biomaterials encompasses a wide range of chemical compositions and bulk physical properties, since they can be prepared from a vast library of available monomers and crosslinkers [2]. Hydrogels can encapsulate and protect hydrophilic drugs from hostile environments with a resultant slow and controlled release by diffusion or erosion [3,4,5]. Multiple studies have demonstrated the applicability of hydrogels for the release of a broad range of therapeutic agents including growth factors, DNA, small interfering ribonucleic acid (siRNA), and anti-cancer drugs [6,7,8,9,10]. 

Hydrogel that can be administered through minimally invasive delivery is an attractive proposition that can enhance patient convenience, ease surgical administration, and reduce costs associated with surgery [11]. Injectable hydrogels can readily mold into the shape of the surgical cavity and adhere to the surrounding tissues during hydrogel formation, which facilitates treatment of any non-standard geometry [3]. The therapeutic cargo dissolved within the structure of hydrogel is shielded from injection-associated shear forces and can be released with complex dynamics in a controlled manner [12]. These injectable hydrogels can be prepared using non-toxic chemical crosslinkers, enzymes, or physical interactions, such as hydrophobic and ionic interactions, and can respond to external stimuli (e.g., temperature, pH, and light), conferring “smart” or “intelligent” systems [5,13]. The thermosensitive hydrogel (i.e., responsive to temperature change) is one of the most popular and widely analyzed systems for drug delivery, facilitating an in situ gelation [3]. Poly(*N*-isopropylacrylamide) (PNIPAAm) is one such example these stimuli-responsive polymers. PNIPAAm undergoes a sharp and reversible coil-to-globule phase transition above 32 °C (the low critical solution temperature, LCST) [9,14]. Below this temperature, the hydrogel is hydrophilic as the hydrogen bonding between isopropyl side groups on the polymer and water molecules is dominant. The polymer chains are thus excessively hydrated and the expanded structures form a hydrogel [15]. Above the LCST, PNIPAAm displays a sharp transition phase with a shrinking conformation as a result of interactions between the hydrophobic segments with a concurrent reduction in the polymer-water hydrogen bonding. At this point, the hydrogel is hydrophobic and the chains rapidly dehydrate and aggregate to form a viscous gel [16]. This temperature-dependent reversible reaction with the LCST close to body temperature has resulted in the application of PNIPAAm hydrogels for drug delivery [17,18]. Grafting PNIPAAm chains onto the backbone of natural polymers (e.g., chitosan, collagen, alginate, and hyaluronic acid) is a strategy that ensures biodegradation, flexibility of gelling temperature, and manipulation of the mechanical properties in hydrogels [19,20]. 

Chitosan (Cs) can be employed as a drug delivery copolymer and has many advantages, including biocompatibility, non-toxicity, bioactivity, biodegradability, low cost, and ease of modification through hydroxyl and amino groups [19]. Cs and derivatives have been utilized as delivery vehicles in various forms, such as film, hydrogel, and microsphere for delivery of vaccines, DNA, and insulin [21,22,23,24]. The hydrogel and nanoparticles composed of Cs backbone grafted with PNIPAAm (Cs-g-PNIPAAm) have been reported as non-cytotoxic with improved drug loading efficiency compared to PNIPAAm alone [16,18,25,26]. Chuang et al. fabricated Cs-g-PNIPAM porous nanoparticles for the encapsulation of a hydrophilic drug (doxycycline hyclate) and reported continuous in vitro release over 10 days [18]. In addition, Cs-g-PNIPAAm copolymers polymerized by free radical copolymerization using ammonium persulfate (APS) and *N*,*N*-methylenebisacrylamide resulted in a dual responsive targeted drug delivery system of magnetic nanoparticles with drug loading of 86% followed by a 70% drug release profile over 10 min [25]. PNIPAAm-based hydrogels can also be utilized as carriers for nucleic acid therapies as the affinity of PNIPAAm copolymers to DNA and the transfection efficiency of delivered DNA can be controlled via temperature change [27]. Trimethyl Cs (TMCs)-g-PNIPAAm has been demonstrated as an effective thermoresponsive gene carrier with 80% transfection efficiency and minimal cytotoxicity in human embryonic kidney cells, HEK293 [16]. However, nucleic acids often require vectors to protect from degradation in the physiological environment and to augment transport across the plasma membrane and facilitate endosomal escape. A 30-mer cationic amphipathic peptide known as RALA (i.e., WEARLARALARALARHLARALARALRACEA) has been successfully employed as a non-viral system for successful gene delivery [28]. RALA condenses nucleic acids through spontaneous electrostatic self-assembly to form cationic nano-sized complexes with the physiochemical properties required for cellular delivery [29]. The rise in utilizing copolymers as delivery platforms for therapeutic nanoparticles has led to the need for a greater understanding of structure-property relationships between hydrogel and therapeutic cargo. This study was designed to characterize the structure-property relationship of Cs-g-PNIPAAm hydrogels where dissolution media and Cs components are considered in the context of how the physiochemical properties relate to successful localized gene delivery.

Simple free radical polymerization was employed to conduct the grafting of PNIPAAm onto a Cs backbone as reported by Pentlavalli et al. [14]. Radical polymerization is a swift and effective way to produce grafted copolymers with unsaturated monomers such as NIPAAm monomers. It negates the requirement for regioselective activation and/or protection of functional groups of the Cs as would be required for condensation methods of polymerization. Ammonium persulfate (APS) and tetramethyl-ethylenediamine (TEMED) initialize the formation of primary radicals, as previously described by Feng [30]. The free radical polymerization avoids multistep synthesis and associated toxicity issues offering a simple, cyto-compatible, scalable, and cost-effective copolymer fabrication [14].

In this study, the structure, morphology, thermoresponsive properties, and in vitro drug release of electrostatic self-assembled nanoparticles were examined. The effect of differing the ratio of Cs in relation to PNIPAAm on the properties of copolymer was also interrogated. Cytotoxicity, swelling behavior, and drug release profiles in different media (i.e., water and Tris-ethylenediaminetetraacetic acid (EDTA) buffer) was also measured. To explore tunable swelling and release properties of the hydrogel, the Cs-g-PNIPAAm copolymer was crosslinked with genipin, a water soluble, non-toxic crosslinker that can undergo self-polymerization [5].

## 2. Materials and Methods

### 2.1. Materials

*N*-Isopropylacrylamide (NIPAAm) with purity ≥99%, low molecular weight Cs with a degree of acetylation of 75–85%, and molecular weight (MW) of 50-190 kDa based on viscosity, APS, TEMED, and genipin were purchased from Sigma-Aldrich, UK. Double distilled water (DDW) was used for copolymer preparation, and the hydrogels were purified by using the membrane filter, Spectra/Por^®^ molecular porous membrane tubing with cut-off 10,000 Da, purchased from Spectrum Laboratories, Inc. (Rancho Dominguez, CA, USA). The RALA peptide (peptide sequence WEARLARALARALARHLARALARALRACEA) was supplied as a lyophilized powder from Biomatik Corporation (Wilmington, DE, USA). The powder was stored at −20 °C and reconstituted in DNase/RNase free water (Life Technologies, Paisley, UK) for experimentation. A plasmid encoding the green fluorescent protein (GFP), pEGFP-N1, Clontech, Mountain View, CA, USA was propagated in MAX Efficiency^®^ DH5α™ Competent Cells (Life Technologies, Paisley, UK), purified using PureLink^®^ HiPure Plasmid Filter Maxiprep Kit (Life Technologies, Paisley, UK), and quantified by UV absorption at 260 nm. NCTC-929 murine fibroblast cell lines were obtained from ATCC, USA. Cells were cultured and maintained in Minimum Essential Medium (MEM) (Gibco, Life Technologies, Paisley, UK) supplemented with 10% horse serum (HS) (Life Technologies, Paisley, UK) in a humidified incubator at 37 °C and 5% CO_2_. Prior to use, cells were trypsinized using trypsin/EDTA in phosphate buffer solution (PBS) (2X) (Life Technologies, Paisley, UK), centrifuged, and resuspended in MEM. Cytotoxicity of the degraded hydrogel product was measured using CellTiter 96 Aqueous One Solution Cell Proliferation Assay (MTS) (Promega, Southampton, UK). Tris-EDTA buffer (1X) was purchased from Thermo Fisher, Abingdon, UK. Quant-iT™ PicoGreen^®^ Reagent was purchased from Life Technologies, Paisley, UK.

### 2.2. Preparation of Cs-g-PNIPAAm

**Figure 1:**
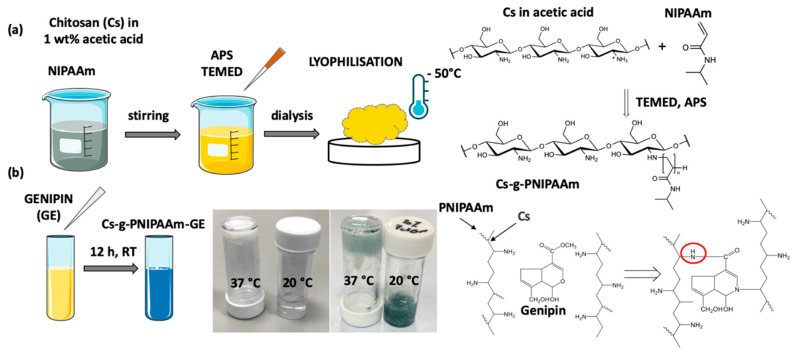
(**a**) Schematic of Chitosan-grafted-Poly(NIPAAm) synthesis. (**b**) Crosslinking of Cs-g-PNIPAAm with genipin (GE).

Cs-g-PNIPAAm, with 10, 20, and 30 wt% of Cs in relation to PNIPAAm was synthesized through free radical polymerization: NIPAAm was added to 1% weight/volume (*w*/*v*) Cs in acetic acid. Solution was purged with dry nitrogen for 60 min prior to polymerization to remove any oxygen in order to avoid a reaction with free radicals, followed by addition of 0.131 mmol initiator APS and 0.2 mmol accelerator TEMED. Copolymerization was achieved within 12 h under ambient conditions. The copolymer was dialyzed for 5 days in DDW at 20 °C to remove any unreacted monomers and recovered by freeze-drying at −50 °C (Figure 1a). Lyophilized copolymer was than solubilized by mechanical agitation in DDW, Tris-EDTA buffer, or PBS at a 5% *w*/*v* ratio for 18 h. For the crosslinked samples, genipin was dissolved in 60% ethanol to 1 mg/mL solution and added to the reconstituted hydrogel at 1% *w*/*v* (Figure 1b). Genipin reacts with Cs producing a blue-colored fluorescent sample, as shown in Figure 1b [31].

### 2.3. Synthesis of Nano-Complexes

The RALA/pEGFP-N1 nanoparticles (NPs) were prepared at a N:P ratio of 10 (the molar ratio of positively charged nitrogen atoms in the peptide to negatively charged phosphates in the pEGFP-N1 backbone) by adding appropriate volumes of RALA peptide solution to 1 μg pEGFP-N1. The complexes were incubated at ambient temperature for 30 min to allow the formation of the NPs.

To visualize the incorporation of the NPs into the hydrogel matrix using scanning electron microscopy [32], representative RALA/hydroxyapatite (HA) NPs were used as the nano-sized HA can be easily observed using scanning electron microscopy [33]. The nano-complexes were formulated at a mass ratio 7:1 via electrostatic attraction as previously described [34]. Briefly, negatively charged HA was incubated with the positively charged RALA peptide for 30 min at 20 °C. The resultant NPs had an average diameter of 153.7 ± 18.9 nm and ζ-potential of 24.8 ± 5.4 mV.

All NPs were lyophilized with 5% *w*/*v* trehalose as a cryoprotectant using VirTis Wizard 2.0 (SP Scientific, Ulster, NY, USA) and stored at ambient temperature.

### 2.4. Chemical Characterization of Cs-g-PNIPAAm Hydrogel

(1) Nuclear magnetic resonance analysis

Proton nuclear magnetic resonance (^1^H-NMR) spectra were recorded on a Bruker 400 plus spectrometer (Bruker Ltd., Durham, UK) in deuterium oxide (D_2_O) at a frequency of 400 MHz.

(2) Fourier-transform infrared spectroscopy

Fourier transform infrared spectra were obtained using Jasco FT/IR 4100 (Jasco Ltd., Dunmow, UK) to determine the copolymer formation. Samples were scanned in a spectral range of 400–4000 cm^−1^ at 256 scans per spectrum and a resolution of 4 cm^−1^.

(3) Thermogravimetric analysis

Thermogravimetric Analysis (TGA) of Cs-g-PNIPAAm copolymers was performed using a TGA Q500 analyzer (TA Instruments, New Castle, DE, USA). Lyophilized samples weighing approximately 8–10 mg of were placed on a platinum pan and heated at a rate of 10 °C min^−1^ from 20 to 600 °C in a nitrogen atmosphere at a flow rate of 40 mL/min for the balance and 60 mL/min for the sample. The loss of copolymer weight was measured and the onset temperature was calculated.

The grafting efficiency was calculated using Equation (1):

(1) $$\mathrm{Grafting}\;\mathrm{efficiency}\;(\%)=\frac{\mathrm{mass}\;\mathrm{of}\;\mathrm{graft}\;\mathrm{copolymer}}{\mathrm{mass}\;\mathrm{of}\;\mathrm{NIPAAm}\;+\;\mathrm{mass}\;\mathrm{of}\;\mathrm{chitosan}}\;\times \;100\%.$$

(4) Zeta potential analysis

Cs-g-PNIPAAm with 10, 20, and 30% of Cs component was reconstituted in DDW, Tris-EDTA buffer, or PBS at a concentration of 2.5% *w*/*v*. Fifty µL of each copolymer and Cs in 1% acetic acid were mixed with 950 µL of ultrapure H_2_O and the ζ-potential was analyzed using a Nano ZS Zetasizer and DTS software (version 7.11, Malvern Instruments, UK).

### 2.5. Rheological Analysis

Rheological properties of 5% *w*/*v* hydrogels in DDW, Tris-EDTA buffer, or PBS were tested using a stress-controlled rheometer model AR-2000ex (TA Instruments, New Castle, DE, USA) equipped with a parallel plate geometry (40 mm diameter). Then, 1.5 mL of the solubilized hydrogel was placed on the rheometer base and analyzed through oscillation mode. Measurements were performed in the linear viscoelasticity region of each sample. The system was equilibrated for 120 s before each measurement and a water trap was used during data acquisition to minimize evaporation.

A time-dependent experiment was carried out at a frequency of 1 Hz for 20 min at 37 °C, measuring the storage modulus (G′) and loss modulus (G″) to determine stability of the hydrogel. Additionally, the viscosity was measured in a flow mode at 20 °C through continuous ramp with shear rate from 0 to 100 s^−1^ for 10 min. The G′ and G″ were measured through the temperature sweep procedure at a frequency of 1 Hz over the temperature from 20 to 50 °C at a rate of 3 °C/min. The LCST was determined as the temperature at which tan δ ≤ 1, where tan δ = G″/G′.

### 2.6. Scanning Electron Microscopy (SEM)

Lyophilized copolymers were individually solubilized in RALA-HA NPs (HA concentration 50 µg/mL) solution or HA-only solution (50 µg/mL) to a concentration of 5% *w*/*v* under agitation for 18 h at 4 °C. The hydrogels ± NPs were lyophilized prior to analysis to remove water content. The analysis was conducted using a JEOL 6500 FEG SEM (Advanced Microbeam Inc., Vienna, OH, USA) at an operational voltage of 3.0 kV. Samples were fixed onto aluminum discs and sputter-coated with gold to avoid electrical charging.

### 2.7. Swelling Ratio of the Cs-g-PNIPAAm Hydrogels

Swelling behavior was measured by solubilizing 25 mg of each lyophilized copolymer with 1 mL DDW, Tris-EDTA buffer, or PBS at 4 °C for 18 h. Hydrogels were then solidified at 37 °C for 1 h. After a defined time interval, excess DDW, TE, or PBS was removed carefully from each well to ensure the increase in weight was due to the fluid absorption, and mass of swollen hydrogel was measured.

The equilibrium swelling ratio was calculated according to Equation (2).

(2) $$\mathrm{Swelling}\;\mathrm{ratio}\;(\%)=\frac{\left(\mathrm{Wt}-\mathrm{Wo}\right)}{\mathrm{Wo}}\;\times \;100\%$$

where Wo was the weight of initial lyophilized copolymer and Wt was the weight of swollen hydrogel sample.

### 2.8. Injectability Analysis

Solubilized hydrogels (5% *w*/*v* in DDW) were injected into the air through a 25 gauge (25-G) cannulated needle for a distance of 20 mm at a displacement rate of 1 mm/s. The injection force was recorded using a TA.XT plus Tensile Analyzer using Exponent software (Version 6, Godalming, UK).

### 2.9. Indirect Cytotoxicity Assessment

Mouse fibroblast NCTC-929 cells were plated in 96-well plates at 10 × 10^3^ cells/well and incubated for 18 h at 37 °C. Then, 100 mg of the lyophilized Cs-g-PNIPAAm hydrogel was sterilized in an UVA-10 Ultraviolet Transilluminator (Ultra-Lum, Claremont, CA, USA) at a wavenumber of 365 nm for 16 h at maximum intensity. Sterilized copolymer was incubated in 2 mL of MEM cell culture media for 48 h at 37 °C. Supernatant was withdrawn and stored at 4 °C and the extract concentration was calculated as 1.2 mg/mL.

NCTC-929 cells were transfected with 100 μL of residual product (120 µg/well) for 4 h, followed by PBS wash and replenishment with normal culture medium. Extract from high-density polyethylene (HDPE) sheet (10 mm × 20 mm) was incubated in 2 mL of MEM cell culture media at 37 °C for 48 h and 10% DMSO in MEM was used as negative and positive control as per ISO 10993-5 [35]. Cell viability was assessed 24 h post-transfection with CellTiter 96^®^Aqueous One Solution Cell Proliferation Assay using manufacturer’s protocol, and absorbance was measured at 490 nm using a FLUOstar Omega Microplate Reader (BMG Labtech, Aylesbury, UK). Cell viability of each treatment was normalized to an untreated control group.

### 2.10. RALA/pEGFP-N1 NP Delivery from Cs-g-PNIPAAm Hydrogel

The lyophilized Cs-g-PNIPAAm copolymers were solubilized to 5% *w*/*v* concentration with RALA/pEGFP-N1 NPs’ suspension for 18 h at 4 °C (pEGFP-N1 concentration in hydrogel was equivalent to 33.3 μg/mL). Then, 300 μL of each hydrogel containing 10 µg of pEGFP-N1 were formed into discs by incubating at 37 °C for one hour in individual wells of 24-well plate. Then, 1.5 mL of DDW was added to each well containing hydrogel disc and stored at 37 °C. At defined time intervals, three supernatant samples of 50 μL were withdrawn and stored at ambient temperature. To measure pEGFP-N1 release in supernatants at each time point, supernatant samples were analyzed via Quant-iT™ Picogreen assay (Life Technologies, Paisley, UK). Percentage DNA release was calculated using a standard curve between concentrations of 0.1 and 10 μg/mL. Then, 50 μL of each sample was placed in individual wells of a black, 96-well plate. To dissociate complexed RALA/ pEGFP-N1 NPs, 50 μL of 1 µg/µL Proteinase K (Sigma Aldrich, Dorset, UK) was added to the NPs’ suspension and incubated for 120 min at 37 °C. Finally, 50 μL of diluted Picogreen reagent was added to each well. Samples were excited at a wavelength of 480 nm and the fluorescence emission was measured at 520 nm using a FLUOstar Omega Microplate Reader (BMG Labtech, Aylesbury, UK).

To assess the DNA transfection efficiency following release from the hydrogel, the supernatant containing released NPs was collected at a 24-h time point. Then, 15 × 10^3^ NCTC-929 cells were plated into a 96-well plate and incubated for 18 h at 37 °C, followed by incubation in Opti-MEM media for 2 h at 37 °C. The supernatant containing RALA/pEGFP-N1 NPs released from the hydrogel was transferred to a well plate for a direct contact with cells. The plate was incubated for 6 h at 37 °C followed by replacing the supernatant with complete MEM media. Images were taken 48 h post-transfection, using an EVOS FL fluorescent microscope (Life Technologies, Paisley UK). Samples were prepared for measurement of transfection efficiency using fluorescence-activated cell sorting (FACS) flow cytometry. The percentage of GFP-expressing cells was detected by FACS Calibur system (BDBiosciences^®^, Berkshire, UK), fixing fluorescence gating at 1.5%.

### 2.11. Statistical Analysis

The difference between the groups was performed using one-way or two-way ANOVA (analysis of variance) with Bonferroni post hoc test using GraphPad Prism version 8 (GraphPad, San Diego, CA, USA). Results represent the mean of three independent repeats (n = 3) ± SEM, unless otherwise stated. A statistically significant difference was considered when p-value was 0.05 or less, (*) p ≤ 0.05, (**) p ≤ 0.01, (***) p ≤ 0.001).

## 3. Results

**Figure 2:**
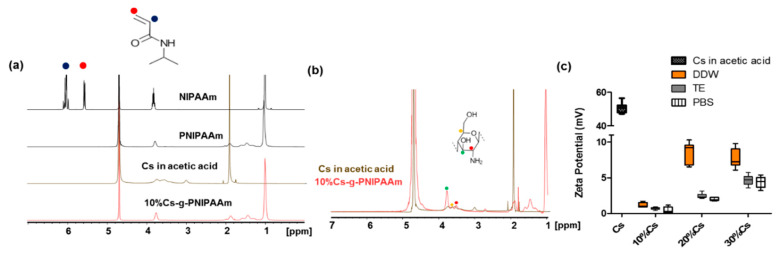
Verification of Cs-g-PNIPAAm composition via (**a**) ^1^H-NMR spectra of NIPAAm monomer, PNIPAAm, Chitosan in 1% acetic acid, and Cs-g-PNIPAAm copolymer; (**b**) comparison of Cs in acetic acid and Cs-g-PNIPAAm copolymer. ^1^H-NMR confirmed presence of PNIPAAm and chitosan in the structure. Lack of peaks between 5.5–6.5 ppm demonstrates no monomers in the copolymer. (**c**) The ζ-potential of Cs and Cs-g-PNIPAAm with 10%, 20%, and 30% Cs in relation to PNIPAAm after reconstitution in different media (DDW, Tris-EDTA (TE) buffer, PBS).

Cs-g-PNIPAAm copolymers were formulated with 10, 20, or 30% of Cs in relation to NIPAAm monomers. The 10% Cs-g-PNIPAAm composition was assessed via ^1^H-NMR (Figure 2a). Spectra of NIPAAm monomer, PNIPAAm, and 10% Cs-g-PNIPAAm exhibited peaks at 1 ppm originated from the methyl group (–CH_3_) and at 3.7 ppm attributed to isopropyl (–CH) signal. The intensity of the two broad peaks of ethyl groups (–CH_2_–CH) at 1.4 and 1.9 ppm observed in PNIPAAm and 10% Cs-g-PNIPAAm were enhanced due to the presence of the methyl group of PNIPAAm component [36]. The peak at 4.7 ppm originated from the D_2_O solvent [9]. The double-bond peaks of the NIPAAm monomer observed at 5.7 ppm and 6.1 ppm were not present in PNIPAAm and 10% Cs-g-PNIPAAm, indicating successful polymerization of NIPAAm [37]. The ^1^H-NMR analysis confirmed the presence of both PNIPAAm and Cs in the molecular structure. At higher magnification (Figure 2b) peaks between 3 and 4 ppm were assignable to protons of C-3, C-4, C-5, and C-6 of the Cs pyranose ring [38,39]. Two reactive groups in the Cs backbone were used for grafting, the amine groups of the deacetylated units on C-2 carbons and C-3 and C-6 hydroxyl groups of acetylated or deacetylated units of Cs component [40]. Due to the higher reactivity of C-2 compared to hydroxyl groups at C-3 and C-6 on the chitosan backbone, most grafting reactions occurred at C-2 [39]. In agreement with other studies [41], the lack of ^1^H-NMR spectra associated with C-2 in the pyranose ring of Cs indicated that this site was used for grafting in the 10% Cs-g-PNIPAAm.

Cs in 1% acetic acid exhibited a high positive ζ-potential of 50.6 mV ± 8.6. The 10% Cs-g-PNIPAAm was close to neutral for DDW, Tris-EDTA buffer, and PBS. Increasing the Cs component to 20% in relation to NIPAAm increased the ζ-potential in all media. Increasing the Cs content to 30% resulted in a surge when the hydrogel was reconstituted in Tris-EDTA buffer and PBS, but no increase was observed when DDW was used (Figure 2c). Cs is a cationic polyelectrolyte [42], which was confirmed by the ζ-potential analysis (Figure 2c). The ζ-potential of 10% Cs-g-PNIPAAm in all media (DDW, TE, and PBS) was close to 0, which suggests that all charged amino groups have been utilized during the polymerization with NIPAAm. The excess Cs resulted in a higher ζ-potential of 20% and 30% Cs formulations, particularly in DDW due to the higher relative acidity of amino group with pKa of 6.5 in DDW (pH 7) compared to PBS (pH 7.4) or Tris-EDTA buffer (pH 8) [43].

**Figure 3:**
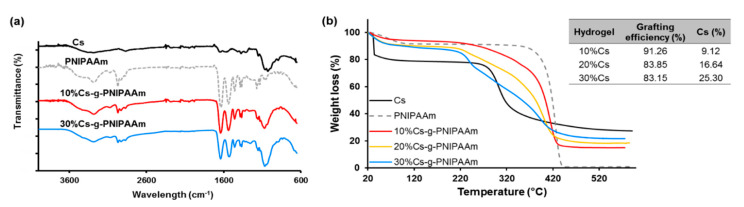
(**a**) FTIR confirming presence of the individual component in the copolymer, (**b**) grafting of the copolymers analyzed via TGA: Representative degradation profiles of hydrogel with varied Cs% showed two transition phases (typical of pure Cs and PNIPAAm). Mass remaining at 600 °C was attributed to Cs component and the mass % of Cs component from TGA corresponded to Cs utilized during hydrogel synthesis. Insert shows grafting efficiency and Cs context calculated with weight remaining at 600 °C.

The characterization of Cs-g-PNIPAAm copolymers via FTIR confirmed the presence of PNIPAAm and Cs (Figure 3a). The amine stretching N–H of PNIPAAm was detected as forming a peak at 3280 nm [44]. The carbonyl stretching vibration of amide I on PNIPAAm was detected at 1681 cm^−1^ and N–H bending vibration (amide II) at 1535 cm^−1^ [45]. The isopropyl group of PNIPAAm formed peaks at 2973 cm^−1^ and 1173 cm^−1^ from the –CH group, and a doublet peak at 1365 cm^−1^ and 1369 cm^−1^ from the –CH_3_ group. The bands at 1062 and 1002 cm^−1^ correspond to C–O stretching in Cs [46,47], confirming presence in copolymers. The typical bands of N-acetyl groups at around 1645 cm^−1^ (C=O stretching of amide I) and 1325 cm^−1^ (C–N stretching of amide III), respectively, present in the Cs sample were masked by the high concentration of PNIPAAm, which showed peaks in the same region [48]. The graft copolymers showed the typical bands for both PNIPAAm and Cs [41]. The intensity of –CH_3_ peak and –CH group on copolymers peaks decreased compared with pure PNIPAAm. In addition, the relative signals of Cs increased with respect to those of PNIPAAm as the Cs component increased from 10 to 30%.

TGA analysis confirmed the successful grafting of the copolymers. The thermal degradation of Cs-g-PNIPAAm copolymers with 10, 20, and 30% of Cs in relation to NIPAAm was compared to that of the constituents (Figure 3b). Cs and PNIPAAm displayed two distinctive degradation steps with PNIPAAm showing higher thermal stability than Cs, until complete degradation at 445 °C. The thermogram of Cs exhibited two transition phases. The first occurred at 38 °C due to loss of water molecules [48] with a weight loss of about 14%. The primary degradation started at 280 °C and was due to dehydration of sugar rings, depolymerization, and decomposition of acetylated and deacetylated units [41]. Degradation of PNIPAAm was confined to a single degradation step at 371 °C. The copolymer containing 10% Cs in relation to PNIPAAm exhibited an intermediate weight loss compared to that of the pristine PNIPAAm. A distinct two-step degradation process was observed for the 20% Cs-g-PNIPAAm and 30% Cs-g-PNIPAAm hydrogels. The onset of the first major weight loss at 220 °C and 227 °C for hydrogels containing 20% and 30% Cs corresponded to the decomposition of the main chains of the Cs. The second major weight loss commenced at 350 °C and 362 °C and corresponded to the decomposition of the grafted PNIPAAm component. A clear Cs degradation step was not observed for the 10% Cs-g-PNIPAAm hydrogel and only a degradation step relating to the PNIPAAm component was observed at 340 °C. The difference in percentage remaining weight at 600 °C was indicative of the different Cs content in the formulations. The copolymer with 10% Cs had a remaining weight of 15% at 600 °C, whereas 20% and 30% Cs-g-PNIPAAm had a remaining weight of 18.58% and 21.73%. This confirmed the presence of Cs in all copolymers, as PNIPAAm was completely decomposed at this temperature. Based on the percentage of remaining weight and initial weight, the Cs content and grafting efficiency were calculated (Table insert in Figure 3b). The grafting efficiency decreased with an increase in Cs content. The decrease in grafting efficiency reduced the thermal stability from 280 °C for Cs to 220 °C and 227 °C for the 20% and 30% Cs-g-PNIPAAm. The reduction in thermal stability was attributed to the PNIPAAm affecting the crystalline region of Cs [42,46]. Fortunately, these thermal changes took place at physiologically irrelevant temperatures that would never be experienced in vivo.

**Figure 4:**
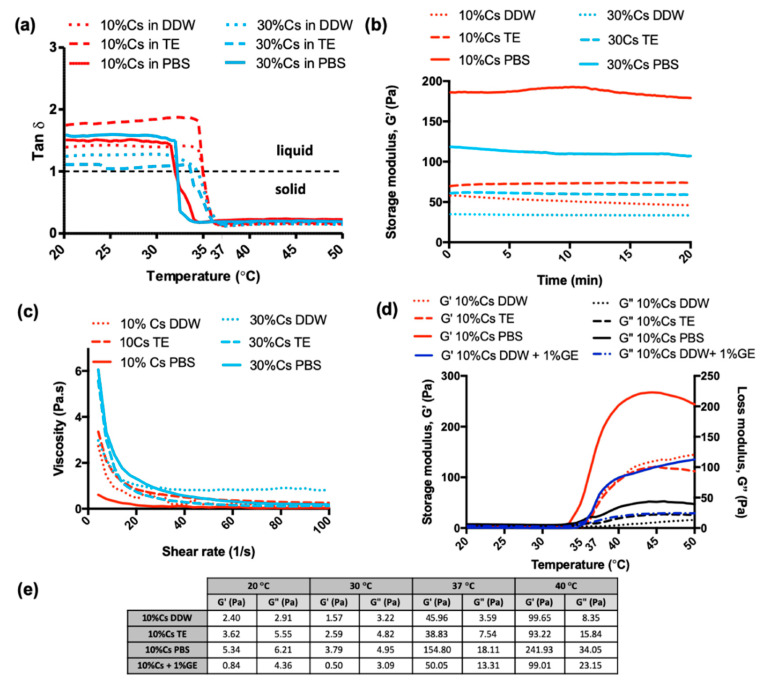
Rheological characterization of Cs-g-PNIPAAm: (**a**) Tan δ of gel as a function of temperature achieved using ramp temperature analysis, where the tan δ value shifted from a value > 1 to a value < 1. Dashed line represents equilibrium state of the gel when liquid solution and solid gel phases were equal (G′ = G″). (**b**) Hydrogel stability assessed via time sweep at 37 °C over a 20 min period. (**c**) Viscosity of gels in liquid state at 20 °C. (**d**) Dynamic temperature sweep of Cs-g-PNIPAAm copolymers in PBS, DDW, and Tris-EDTA (TE) buffer demonstrating ability to modulate mechanical properties while maintaining consistent sol-gel transition point. (**e**) Shear storage modulus (G’) and shear loss modulus (G”) values extracted from the dynamic temperature sweep.

Rheological properties of Cs-g-PNIPAAm hydrogels were studied to compare the effect of the Cs content and reconstitution media on the viscoelastic properties. The values for G′ and G″ were monitored as a function of temperature (Figure 4a). The tan δ was calculated as G″/G′. All compositions were expected to show thermoresponsive behavior due to the presence of PNIPAAm [26] with a shift from tan δ > 1 to tan δ < 1, indicating the transition from a liquid to a solid at the LCST [14]. Figure 4a indicates the LCST of the Cs-g-PNIPAAm gels depended upon the dissolution fluid. Samples solubilized in PBS exhibited a LCST of 32 °C irrespective of the Cs content. The LCST for the Cs-g-PNIPAAm gels in Tris-EDTA buffer was 34 °C and 34.5 °C as a function of 10% and 30% Cs. Similar differences were observed in samples solubilized in DDW, i.e., 35 °C for the Cs-g-PNIPAAm gel containing 10% Cs and 34.5 °C for the 30% Cs equivalent. All hydrogels were stable over time, exhibiting a constant tan δ < 1 when subjected to a mechanical oscillation frequency of 1 Hz for up to 20 min at 37 °C (Figure 4b). The value for G’, which is conceptually similar to the Young’s modulus, depended upon the dissolution media: 10% Cs-g-PNIPAAm exhibited a G’ of 154.8 Pa ± 49.5 when solubilized in PBS, which was significantly higher compared to 46.51 Pa ± 31.1 and 38.3 Pa ± 15, when solubilized in Tris-EDTA buffer and DDW. At 37 °C, the G’ of 10% Cs-g-PNIPAAm crosslinked with 1% genipin was 50 Pa ± 4.8, which was higher than non-crosslinked samples in DDW and TE. Crosslinking had no effect on the LCST (35 °C).

**Figure 5:**
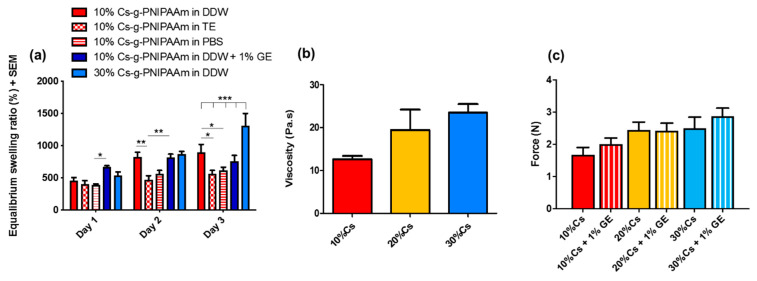
(**a**) Swelling behavior of Cs-g-PNIPAAm hydrogels in different media (PBS, DDW, and Tris-EDTA (TE) buffer). (**b**) Viscosity of the hydrogels determined in a flow mode at 20 °C. (**c**) Force required to inject 200 µL hydrogel through 25-G needle and viscosity of copolymers measured via rheological measurement in flow mode.

Viscosity measurement at 20 °C (Figure 4c and Figure 5b) indicated that 30% Cs-g-PNIPAAm hydrogel was significantly more viscous than 10% Cs-g-PNIPAAm. However, the viscosity of the 20% Cs-g-PNIPAAm hydrogel was not significantly different to the same hydrogel systems containing 10% or 30% Cs. Crosslinking with 1% genipin did not increase the viscosity of hydrogels at ambient temperature.

There was no significant impact of Cs content on the swelling ratio of the hydrogels in DDW at Day 1 and Day 2 (Figure 5a). The swollen hydrogels exhibited a mass range of 457–873% of dry weight due to DDW intake. At Day 3, the 10% Cs-g-PNIPAAm hydrogel demonstrated a significantly higher swelling ratio in DDW compared to PBS or Tris-EDTA buffer. The hydrogel in PBS showed the lowest degree of swelling at all time points. The 1% *w*/*v* genipin crosslinking did not affect the swelling behavior of 10% Cs-g-PNIPAAm in DDW. The 10% Cs hydrogel showed significantly lower swelling behavior in TE compared to DDW at Day 2 and Day 3 (Figure 5a). All hydrogel formulation, except for 30% Cs-g-PNIPAAm, achieved swelling equilibrium in Day 2 and no further swelling was observed in Day 3.

The maximal injection force of the hydrogel formulations was measured following injection in air through a 25-G needle with nominal inner diameter of 0.260 mm. The force required to inject each hydrogel formulation correlated to Cs content (Figure 5b). Crosslinking with 1% genipin did not affect the injectability of the hydrogel at ambient temperature as there was no significant difference in the viscosity of the hydrogel formulations.

**Figure 6:**
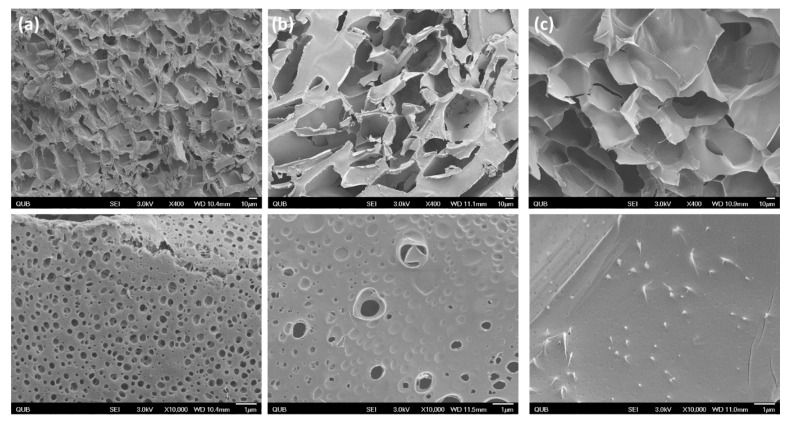
SEM of hydrogel at ×400 (above) and ×10,000 (below): (**a**) Cs-g-PNIPAAm hydrogel, (**b**) Cs-g-PNIPAAm hydrogel with pure HA and, (**c**) Cs-g-PNIPAAm hydrogel with RALA-HA NPs.

The structures of various lyophilized copolymers are presented in Figure 6. The 10% Cs-g-PNIPAAm formed a highly connected and meshed network. Pores observed in the pristine hydrogel (Figure 6a, bottom row) were uniform in shape with an average diameter of 0.48 μm ± 0.09. The presence of HA particles altered the structure of the 10% Cs-g-PNIPAAm as the number of micro-sized pores visible were visibly reduced. Conversely, hydrogels with RALA-complexed HA did not exhibit micropores within the hydrogel structure.

**Figure 7:**
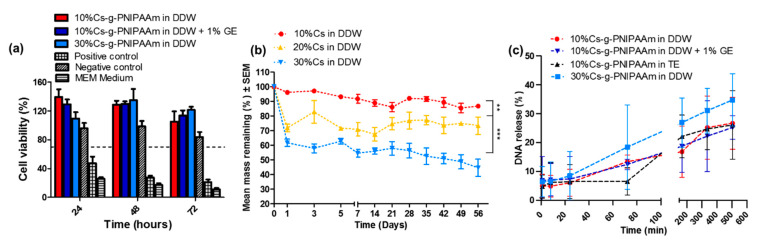
(**a**) Cell viability analyzed via MTS assay for NCTC L929 fibroblasts co-cultured for 24 h with the hydrogel degradation products for 10%, 10% crosslinked with GE, and 30% Cs in relation to NIPAAm. The dashed line delineates 70% viability, below which is deemed cytotoxic, as designated by the ISO 10993–5 [35]. (**b**) Degradation profile of Cs-g-PNIPAAm in DDW over eight weeks. (**c**) Release profiles of RALA/pEGFP-N1 NPs) from 5% *w*/*v* 10% Cs and 30% Cs-g-PNIPAAm hydrogels in DDW at 37 °C. Values represent mean ±SEM (n = 3).

Degradation of the hydrogel was conducted as per ISO 10993-5 [35] and the degradative by-products were placed in contact with NCTC-929 cells for up to 72 h to examine the cytotoxicity. No cytotoxic effects from 1.2 mg/mL Cs-g-PNIPAAm hydrogel residues were observed, with cell viability treated with pure hydrogel and crosslinked formulation higher than that of the untreated control at Day 3 (Figure 7a).

Incubation of pEGFP-N1 with RALA peptide resulted in formation of NPs with a diameter 126.1 nm ± 11.9 and ζ-potential + 18.3 mV ± 4.1. All hydrogels loaded with 10 µg of RALA/pEGFP-N1 NPs provoked a higher release over the initial 8 h, followed by a sustained release (Figure 5c). The RALA/pEGFP-N1 cargo released from the hydrogel within the first 24 h accounted for 6–8.4%, depending on the reconstitution medium and % Cs. At day 21 (504 h) the 30% Cs hydrogel in DDW showed the highest percentage of NPs released, whereas there was no difference between pristine hydrogel and the hydrogel crosslinked with genipin (26.1% compared to 26.6%). Reconstitution of 10% Cs hydrogel in DDW or Tris-EDTA buffer did not affect NP release.

**Figure 8:**
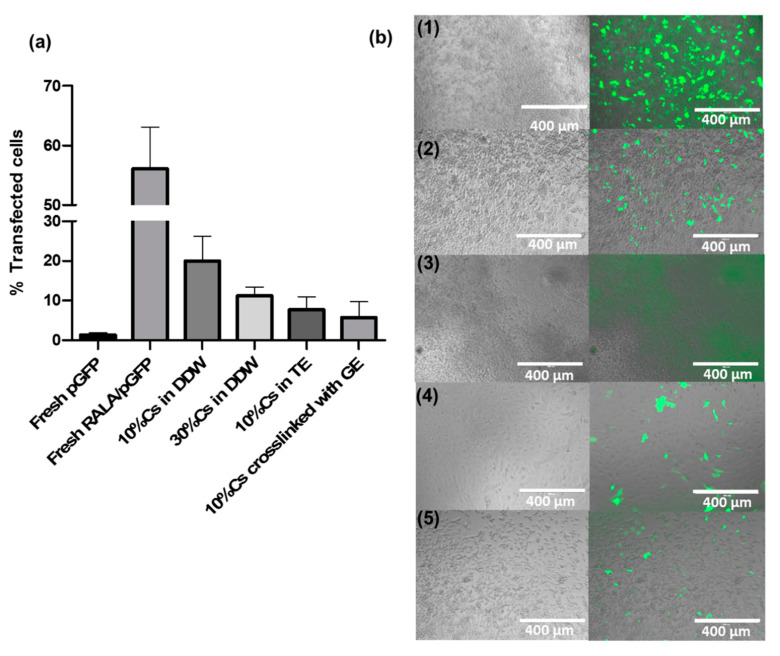
Assessment of DNA transfection efficiency of NCTC-929 cells after incubation with RALA/GFP NPs released from Cs-g-PNIPAAm hydrogels at 24 h time point. (**a**) Flow cytometry analysis showed varied transfection efficiency of RALA/GFP NPs released from the hydrogel formulations. (**b**) Cells transfection efficiency was visualized with the presence of green fluorescent protein expressed by the NCTC-929 cells for (1) fresh NPs, (2) NPs released from 10% Cs-g-PNIPAAm in DDW, (3) NPs released from 30% Cs-g-PNIPAAm in DDW, (4) NPs released from 10% Cs-g-PNIPAAm in Tris-EDTA (TE) buffer, and (5) NPs released from 10% Cs-g-PNIPAAm in DDW crosslinked with 1% GE.

The supernatant containing NPs released from each hydrogel was placed in direct contact with the NCTC-929 cells. Total mass of pEGFP-N1 per wells was calculated as 0.6, 0.84, 0.64, and 0.73 µg for 10% Cs in DDW, 30% Cs in DDW, 10% Cs in TE, and 10% Cs crosslinked with 1% GE, based on the percentage of NPs released (Figure 7c) and the initial loading mass of 10 µg. The efficiency of RALA/pEGFP-N1 NPs to transfect NCTC-929 cells following encapsulation and release from Cs-g-PNIPAAm hydrogel was lower than fresh RALA/pEGFP-N1 NPs in aqueous solution (Figure 8a). Indeed, it decreased from 65.4% for fresh aqueous NPs (0.5 µg per well) to 19.9–5.8% for NPs released from the hydrogel, depending on the formulation. NPs released from 10% Cs-g-PNIPAAm in DDW resulted in the highest transfection efficacy.

## 4. Discussion

Thermoresponsive Cs-g-PNIPAAm hydrogels are typically synthesized via ceric ammonium nitrate [49] or 1-ethyl-3-(3-dimethylaminopropyl) carbodiimide [50]. However, grafting can be problematic with a narrow distribution of molecular weight on the side chains. Free-radical polymerization enables control over molecular weight and polydispersity and is simple, relatively inexpensive [51], and avoids the need for toxic reagents and solvents, which may cause biocompatibility issues for intended in vivo applications [52].

**Figure 9:**
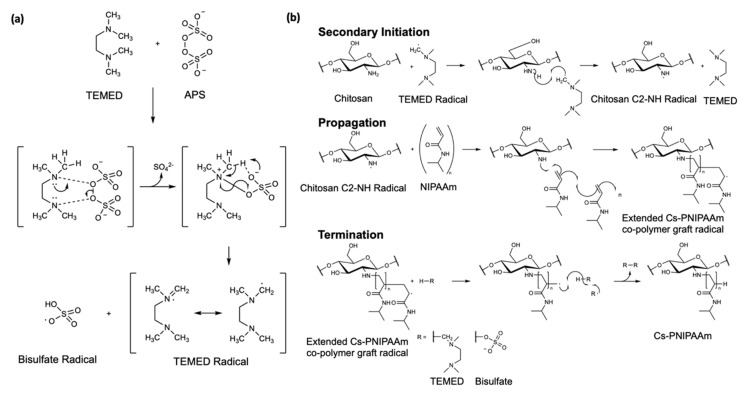
(**a**) Mechanism of primary radical initiation with APS and TEMED. (**b**) Proposed scheme of radial polymerization. Secondary initiation of Cs by TEMED radical, leading to chain reaction propagation with NIPAAm and terminated with a hydrogen atom shift by radical scavenger species (TEMED or bisulfate) to liberate C-2 directed graft of Cs-PNIPAAm copolymer.

The Cs-g-PNIPAAm copolymer in this study was synthesized via free radical polymerization and confirmed via FTIR, ^1^H-NMR in D_2_O, and TGA analysis [53]. It was assumed that the grafting of PNIPAAm chains onto Cs occurred at the carbon C-2 position of the pyranose ring. The formation of amine radicals is highly favored over hydroxyl radicals [54,55], supporting work by Shirangi et al. [56] that demonstrated that primary amines exhibit increased affinities for the α-TEMED radical species (Figure 9a). The aforementioned evidence points to the directed radical formation C-2-NH_2_ species of chitosan [57]. This step also led to the regeneration of TEMED, further consumed in catalytic initiation. The chitosan radical species underwent chain reaction propagation with NIPAAm monomers, as per Figure 9b. The termination step was likely achieved through radical disproportionation where the abstraction of a hydrogen atom by the extended copolymer created an intermediary radical which led to termination. Hydrogen atom abstraction could have occurred from NIPAAm homopolymer but was more likely from the radical initiators [58]. In addition, the higher concentrations of TEMED (0.2 mmol) compared to APS (0.131 mmol) supported the likelihood of increased formation of TEMED radical to facilitate the secondary initiation mechanism (Figure 9b) [56]. In contrast to gel permeation chromatography (GPC) and high-performance liquid chromatography (HPLC), for which retention times do not provide specific detail on the structure of the graft [59], or preparative liquid chromatography–mass spectrometry (LC-MS) methods where mass spectrometry (MS) fragmentation spectra would be the same irrespective of where the NIPAAm chain extension is grafted onto the Cs, the grafting position could be confirmed with the use of ^1^H-NMR in dimethyl sulfoxide (d6-DMSO). The deuterated proton exchange did not occur as in D_2_O, thereby hydroxyl and amine protons were visible on the presented spectrum. The change in the splitting patterns of the hydrogens bonded directly to the C-2 position and a change in integration values, further confirmation of the grafting location, can be provided with correlation spectroscopy (COSY) and nuclear overhauser effect spectroscopy (NOESY) 2D NMR methods, which should form part of a follow-on study [60,61,62,63]. The addition of a Cs backbone altered the stiffness and degradation properties of the gel and the PNIPAAm side chain triggered gelation of the hydrogel at temperatures >32 °C [64]. TGA analysis demonstrated that both Cs and PNIPAAm were incorporated into the structure as the copolymer exhibited typical degradation profiles. The grafting efficiency was calculated from the percentage of Cs in the copolymer compared to the theoretical 10–30% decrease with a decrease in NIPAAm concentration, which is in agreement with Zhang et al., who reported an increase of grafting efficiency in carboxymethyl Cs-PNIPAAm with increase in NIPAAm concentration [13].

Swelling in water is an important characteristic of hydrogels, as the biomaterials with high water absorption could mimic living tissues [65]. As the hydrogel swells, the mesh size increases, which can be exploited to release entrapped drugs through controlled swelling of hydrogels [3]. PNIPAAm has a very high degree of swelling below the LCST, which is associated with its highly hydrophilic side chains [1,66]. The degree of hydrogel swelling is an important parameter controlling the release pattern of loaded drugs from the network [3]. The swelling behavior of 10% Cs-g-PNIPAAm hydrogels in PBS and TE differed from samples in DDW. This difference was associated with the ionic strength of PBS and Tris-EDTA buffer, which is higher compared to DDW [67]. Due to the presence of cationic amino groups on the polymer backbone, Cs exhibits a polyelectrolyte nature upon dissolution [68]. Swelling of hydrogels is attributed to the repulsion of charges along the polymer chains associated with the diffusion of water molecules inside the hydrogel matrix [13]. This theory is supported by significantly higher swelling of the 30% Cs-g-PNIPAAm in DDW compared to 10% Cs-g-PNIPAAm. The ζ-potential of the 30% Cs in DDW is higher when compared to 10% Cs, facilitating a higher degree of repulsion between polymer chains, allowing for water molecules to diffuse. The ions present in PBS affected the charge distribution along the copolymers backbone, reducing the electrostatic repulsion between the positively charged groups of the polymeric chains, leading to swelling in DDW but not in PBS [9]. The ‘charge screening effect’ of the additional cations caused anion-anion electrostatic repulsion and reduced the difference in osmotic pressure between the hydrogel matrix and the reconstitution medium [69]. The high swelling ability of the hydrogels resulted from the low density of the polymer chains, which made the hydrogels mechanically poor [70]. This was in agreement with other findings from this investigation as the 30% Cs-g-PNIPAAm demonstrated higher swelling and lower storage modulus compared to the hydrogel containing 10% Cs. Time sweep rheological analysis at 37 °C (Figure 4b) confirmed that all hydrogels in DDW, TE, and PBS were mechanically stable and could form suitable reservoirs for the long-term delivery of therapeutics in vivo. The 10% Cs-g-PNIPAAm exhibited the highest storage modulus when reconstituted in PBS (Figure 4b). The range of moduli at 37 °C reported in the literature for Cs-PNIPAAm hydrogels varied from approximately 1 Pa [71] to 1.4 kPa [72], depending on the formulation method. Although mechanical strength is not a prerequisite for this application, there should be sufficient strength to remain localized against in vivo biological forces that could disturb or dilute the injected hydrogel so as to provide the therapeutic effect for a required time [70,73]. In vivo results of similarly structured PNIPAAm-hyaluronic acid hydrogels with storage modulus of approximately 5.5 Pa at 37 °C indicated that the mechanical stability of all hydrogel formulations investigated in this study will be sufficient to withstand biological forces in vivo and remain in place once implanted [74].

The most prevalent strategy for a scaffold based on an injectable hydrogel is to develop a free flowing solution that can be easily delivered as a low-viscosity liquid that can subsequently form a solid hydrogel in vivo [75]. The force required to inject all formulations with or without genipin through a 25-G needle was below the maximum injection force of 30 N described by the ISO 11040-4: 2015 [76]. Thus, Cs-g-PNIPAAm hydrogels are highly injectable and can be used as a minimally invasive delivery system. Furthermore, crosslinking with 1% genipin did not increase the viscosity of hydrogels at ambient temperature nor the injectability through a 25-G needle. This gives a robust method for ensuring long-term delivery of a therapeutic cargo.

At ambient temperature, all gels were in a liquid form as G′′ > G′ with tan δ > 1 as the G” was more prominent compared to G’ and dominated the flow properties [9,77,78,79]. As the temperature elevated, the increased thermal movement of the polymer chains resulted in a decreased viscosity and hydrogel formation [14]. At 32 °C, the gel dissolved in PBS and became more elastic as G′ > G′′, and the loss tangent was lower than 1. The temperature of transition from liquid to solid (tan δ change from >1 to <1) was 2.5 °C lower when gel was reconstituted in PBS than in DDW, which is a consequence of the salt-out effect [14,16]. Although increasing the % Cs component resulted in a potential difference in side chain grafting density of PNIPAAm onto Cs, it did not affect the LCST. It can be, therefore, concluded that grafting did not alter the LCST of PNIPAAm, which had the LCST at 32 °C in PBS and 35 °C in water [14], and the hydrophilic/hydrophobic balance of the fabricated hydrogel was unaffected (Figure 4a).

The SEM analysis showed an interconnected porous structure for the 10% Cs-g-PNIPAAm hydrogel, which did not change following NP incorporation. However, adding the HA reduced the number of micro-sized pores within the hydrogel and no micro-sized pores were visible when RALA/HA NPs were used. Although micropores are favorable for water and small molecules’ transport into the hydrogel network, the lack of pores could be advantageous as other studies have indicated that this could prolong drug delivery [80]. Pores can reduce the diffusion length for drug release, leading to rapid release and limited drug loading capacity [3]. Hydrogel encapsulate a drug within a matrix of limited pore size that retards diffusion and releases the drug through hydrolytic degradation. Kang et al. reported pore closure in poly(lactic-co-glycolic acid) (PLGA) to reduce release of proteins [80]. Wang et al. reported that formation of a non-porous film at the surface of PLGA microspheres reduced the permeability and stopped initial burst release of a model peptide drug [81].

To date, PNIPAAm hydrogels have not achieved a clinical acceptance due to the potential cytotoxic effect of NIPAAm [82]. The NIPAAm monomer has shown a concentration-dependent cytotoxicity with endothelial, epithelial, smooth muscle, and fibroblasts [83]. However, the polymerization of NIPAAm resulted in production of a biocompatible PNIPAAm hydrogel [84,85,86,87]. During synthesis of the Cs-g-PNIPAAm hydrogel, any unpolymerized NIPAAm chains were removed during a five-day dialysis purification step using a 10-kDa dialysis membrane to ensure safety. The lack of potential toxic monomer has been confirmed by the ^1^H-NMR analysis. In the present studies, the hydrogel had a positive effect on the proliferation of NCTC-929 cells, which was in agreement with previous studies where a high degree of acetylation in Cs has a stimulatory effect on the fibroblast cells [88].

Cs-g-PNIPAAm hydrogels can be utilized for a controlled, sustained delivery of a variety of nucleic acid-based biotherapeutics, such as microRNA nanoparticles for bone repair [89], RNA-based vaccine vector delivery for melanoma therapy [90], microRNA for cartridge regeneration or to intra-articular delivery of anti-inflammation DNA nanodrug to treat rheumatoid arthritis [91]. The representative plasmid DNA was complexed with a cell penetrating peptide (CPP), RALA, to create stable NPs. Synthesis and characterization of RALA/pEGFP-N1 NPs are well described in the literature [29,92]. The RALA/pEGFP-N1 NPs are electrostatic; therefore, it is imperative that the delivery system is neutral, as the net charge density on hydrogel can result in unwanted interactions between delivery system and the NPs as the electrostatic forces affect the interactions between NPs and hydrogel matrix [93]. The ζ-potential near 0 of the 10% Cs-g-PNIPAAm in DDW and Tris-EDTA buffer was deemed suitable for the delivery of the cationic cargo. All formulations exhibited an initial release of up to 6.6% within 1 h, without a burst release. The NPs entrapped in the hydrogel matrix were released initially by diffusion followed by a combination of diffusion and degradation mechanisms [20]. At day 21, 30% Cs-g-PNIPAAm was capable of delivering a higher percentage of RALA/pEGFP-N1 NPs compared to the remaining samples, which corresponded with the highest degradation rate. This was due to a low grafting density of this formulation, which was evident by high swelling and degradation. This structural configuration was responsible for the higher release of RALA/pEGFP-N1 NPs via diffusion. The polyelectrolyte nature of the Cs component in the hydrogel also influenced the delivery of cationic NPs. The higher positive net charge density of the 30% Cs formulation was expected to interact with the NPs, presumably through weak repulsion forces, further improving the diffusion from the hydrogel matrix. Further experimental work is necessary to establish if the positive net charge density of this formulation interacted with the NPs.

The lack of burst release of the NPs from a polymer delivery system has been attributed to strong interactions between nucleic acid and in situ forming polymethacrylic acid and polyethylene glycol gel, which affected the stability of the plasmid DNA (pDNA) [94]. Nucleic acids are sensitive molecules which can easily be deactivated or unfolded by interactions with the hydrogel matrix [1]. Here the transfection data confirmed the released NPs were functional as the green fluorescence protein was expressed by NCTC-929 cells. However, the RALA/pEGFP-N1 NPs encapsulated in all hydrogel formulations had a lower transfection efficiency when compared to the same NPs in DDW. This may be a consequence of differences in available pDNA present, as the pDNA placed in contact with cells depended on the release and degradation mechanisms. The 30% Cs demonstrated the highest degradation and subsequent release rates. However, the transfection efficiency was lower compared to the 10% Cs, which released 0.6 µg of pDNA released after 24 h, indicating potential interactions of positive net charge density of 30% Cs hydrogel with electrostatic NPs. The highest transfection efficiency of 19.9% for NPs released from 10% Cs further confirmed the suitability of this hydrogel formulation as a drug delivery vehicle. Interestingly, the 10%Cs-g-PNIPAAm in Tris-EDTA buffer and crosslinked with 1% GE showed lower transfection efficiency compared to sample in DDW, despite similar degradation and release rates after 24 h. The interaction of hydrogel with TE dissolution media or genipin might reduce the transfection rate through NP aggregation, which increased the average size of the NPs to above 200 nm, making them too big to be internalized via clathrin-mediated endocytosis by the cells [95]. Higher crosslinking rate should be explored to fully characterize its effect on the properties of the hydrogel such as degradation, rheological properties, porosity, and release mechanism.

## 5. Conclusions

A stable thermosensitive Cs-g-PNIPAAm hydrogel system was successfully synthesized via free radical polymerization. A novel approach of altering the percentage of chitosan in the copolymer was employed to explore its role on properties of the hydrogel. The storage modulus, swelling, degradation, and the level of NP release from the hydrogel depended on the percentage of the chitosan in the copolymer. Furthermore, the viscoelastic properties were tailored by changing the dissolution media. The injectability of the Cs-g-PNIPAAm at ambient temperature suggested potential for minimally invasive delivery to the target site. Sustained NP release and degradation over a three-week period suggested the hydrogel can be utilized as a long-term drug delivery system to the target tissues. The released RALA/pEGFP-N1 were capable of transfecting NCTC-929 cells confirming the hydrogel did not affect the stability of the delivered nucleic acid. Consequently, the synthesized hydrogel formulation can facilitate the long-term drug delivery to the target tissues, as the NPs were readily encapsulated and released by the hydrogels. Future work will focus on long-term release and transfection studies to fully understand the delivery profiles, followed by in vitro and in vivo assessment, which could influence the application of this system in healthcare.
